# SARS-CoV-2 post-acute sequelae linked to inflammation via extracellular vesicles

**DOI:** 10.3389/fimmu.2025.1501666

**Published:** 2025-04-16

**Authors:** Sara Bachiller, Joana Vitallé, Lluís Camprubí-Ferrer, Manuel García, Isabel Gallego, Marina López-García, María Isabel Galvá, Julio Cañizares, Inmaculada Rivas-Jeremías, María Díaz-Mateos, Carmen Gasca-Capote, Cristina Moral-Turón, Lourdes Galán-Villamor, María Fontillón, Salvador Sobrino, José Miguel Cisneros, Luis Fernando López-Cortés, Tomas Deierborg, Ezequiel Ruiz-Mateos

**Affiliations:** ^1^ Institute of Biomedicine of Seville (IBiS), Virgen del Rocio University Hospital, Spanish National Research Council (CSIC), University of Seville, Clinical Unit of Infectious Diseases, Microbiology and Parasitology, Seville, Spain; ^2^ Department of Medical Biochemistry, Molecular Biology and Immunology, School of Medicine, University of Seville, Seville, Spain; ^3^ Experimental Neuroinflammation Laboratory, Department of Experimental Medical Sciences, Lund University, Lund, Sweden; ^4^ Bermejales Physiotherapy Clinic, Seville, Spain; ^5^ Heliopolis Nursing Home, Seville, Spain; ^6^ Pediatric Unit, Virgen Macarena University Hospital, Seville, Spain; ^7^ Service of Pathological Anatomy, Virgen del Rocío University Hospital, Seville, Spain; ^8^ Digestive Service, Virgen del Rocío University Hospital, Seville, Spain

**Keywords:** SARS-CoV-2, post-acute sequelae, extracellular vesicles, immune system, colon tissue

## Abstract

**Background:**

Despite the efficacy of SARS-CoV-2 vaccines in reducing mortality and severe cases of COVID-19, a proportion of survivors experience long-term symptoms, known as post-acute sequelae of SARS-CoV-2 infection (PASC). This study investigates the long-term immunological and neurodegenerative effects associated with extracellular vesicles (EVs) in COVID-19 survivors, 15 months after SARS-CoV-2 infection.

**Methods:**

13 Controls and 20 COVID-19 survivors, 15 months after SARS-CoV-2 infection, were recruited. Pro-inflammatory cytokines were analyzed in both plasma and EVs. A deep-immunophenotyping of monocytes, T-cells and dendritic cells (DCs) was performed, along with immunostainings of SARS-CoV-2 in the colon.

**Results:**

Higher concentrations of pro-inflammatory cytokines and neurofilaments were found in EVs but not in plasma from COVID-19 survivors. Additionally, COVID-19 participants exhibited altered monocyte activation markers and elevated cytokine production upon lipopolysaccharide stimulation. Increased activation markers in CD4+ T-cells and decreased indoleamine 2,3-dioxygenase expression in DCs were observed in COVID-19 participants. Furthermore, the amount of plasmacytoid DCs expressing β7-integrin were higher in COVID-19, potentially associated with the viral persistence observed in the colon.

**Conclusions:**

COVID-19 survivors exhibit long-term immune dysregulation and neurodegeneration, emphasizing the need for ongoing monitoring of PASC. The cargo of EVs can be a promising tool for early detection of virus-induced neurological disorders.

## 1 Introduction

Since the first reported case of coronavirus disease (COVID-19) in 2020 (1), SARS-CoV-2 has rapidly spread, infecting more than 772 million people and causing 6.9 million deaths worldwide (2). Although vaccination has drastically reduced the mortality and the most severe COVID-19 cases (3), 18 months after SARS-CoV-2 infection, 10.4% of COVID-19 survivors present long-term symptoms, known as post-acute sequelae SARS-CoV-2 infection (PASC) or long-COVID (4). Within these symptoms, pulmonary, gastrointestinal, cardiovascular or mental health alterations are the most common (5). In acute infection, people with COVID-19 presented higher levels of pro-inflammatory cytokines (6), exacerbated immune responses and higher levels of neurodegenerative markers in plasma associated to worse clinical outcomes (7), which may predict PASC. In the medium term, immune alterations persist months after SARS-CoV-2 infection regardless of previous hospitalization; however, these alterations are exacerbated by disease severity (8–10), and elderly individuals tend to have a poorer response to COVID-19 vaccines (11–13). No effective treatments exist for the wide spectrum of PASC; therefore, to find the mechanisms behind the heightened inflammation and the altered immune response in PASC is a current necessity.

In this sense, extracellular vesicles (EVs) are widely used to study physiological and pathological cell conditions in plasma samples. Their cargo can be different depending on the functional state of the host cell (14). Related to COVID-19, SARS-CoV-2 nucleocapsid (Nc) protein has been identified in EVs sputum samples from acute SARS-CoV-2 infected people (15). Several proteins involved in immune responses, such as inflammation, activation of the coagulation, and complement pathways, in addition to neurodegeneration-related proteins, have also been found in EVs from acute-infected people (16, 17). However, little is known about the subsequent changes in EVs cargo and associated immune responses related to neurodegeneration in the long term after acute infection. This study aimed to investigate the major immune alterations and neurodegeneration associated with EVs cargo in COVID-19 survivors 15 months after the infection.

## 2 Materials and methods

### 2.1 Study design and participants

Twenty participants were selected from the COVID-19 cohort at Virgen del Rocío University Hospital (VRUH, Seville, Spain) and eighteen Controls (without any documented SARS-CoV-2 infection) from Heliopolis nursing home (Seville, Spain). The Control population included a highly monitored group of elderly individuals, with regulated visits and routine analytical assessments. In fact, due to this rigorous monitoring, five active cases of SARS-CoV-2 were detected during the sample collection and were therefore excluded from the analysis. All the participants underwent the memory alteration test (M@T) (18). Inclusion criteria were: ≥50 years old, confirmed SARS-CoV-2+ PCR (at least 12 months before the M@T), and no SARS-CoV-2 reinfections. Exclusion criteria: drug and alcohol abuse, active infections, hospital admission, anti-tumor therapy, or any immunomodulatory therapy or treatment that could influence the immune system (mainly corticosteroids) at least 6 months before the beginning of the study. Demographic data, self-reported symptoms (COVID-19), and blood samples were obtained at the moment of M@T performance. The COVID-19 and Control groups were age- and sex-matched and had received at least one vaccine dose. Colon biopsies of five COVID-19 participants were collected during a colonoscopy at 30-56-65-90-297 days after SARS-CoV-2+ PCR. Written informed consent was obtained. The study was reviewed and approved by the Ethics Committee of Virgen Macarena and VRUH (C.P. NeuroCOVIH-C.I. 1155-N-21, C.P. S230054-C.I. 1518-N-23).

### 2.2 Cell and plasma isolation

Peripheral Blood Mononuclear Cells (PBMCs) were isolated as previously (11) and cryopreserved (90% Fetal Bovine Serum (FBS), ThermoFisher Scientific, 10% dimethyl sulfoxide, PanReac AppliChem) in liquid nitrogen until further use. Plasma samples were collected as previously (8) and stored at -80°C.

### 2.3 Laboratory methods

Complete hemograms were determined using Epic XL-MCL or FC500 flow cytometer (Beckman Coulter Inc., California). All the assays were performed according to the manufacturers’ instructions.

### 2.4 *In vitro* experiments

For monocyte functional assays, 1x10^6^ PBMCs were *in vitro* stimulated with lipopolysaccharide (LPS, 0.5ng/mL, Invivogen) in R-10 medium (10% FBS, 1% L-glutamine, 1% penicillin-streptomycin in RPMI medium) (5h, 37°C), including a negative control without stimulation. PBMCs were incubated with monensin (Golgi Stop, BD Biosciences) following the manufacturer’s instructions and intracellular cytokines were analyzed by multiparametric flow cytometry.

Flow cytometry.

In general, after washing with PBS (625g, 5min, RT), PBMCs were incubated with the viability marker (LIVE/DEAD Fixable Aqua or LIVE/DEAD Fixable Violet Cell Dead Stain Kits, Invitrogen) and the extracellular antibodies (35min, RT) (**Supplementary Table S1**). PBMCs were then washed, fixed and permeabilized with BD Cytofix/CytoPerm (BD Biosciences) (20min, 4°C) for cytoplasmic markers (Indoleamine 2,3-dioxygenase (IDO) and cytokines) or Fixation/Permeabilization Buffer Set (eBioscience) (45min, 4°C) for Ki67, following the manufacturer’s instructions. Cells were incubated with intracellular markers (30min, 4°C), washed and fixed with 4% paraformaldehyde solution (PFA, Sigma-Aldrich) (20min, 4°C). Extracellular and intracellular markers’ staining was performed adding the volumes/concentrations of each antibody recommended by manufacturers. For monocyte identification, HLA-DR^+^ cells were selected and classical (CD14^++^CD16^-^), intermediate (CD14^++^CD16^+^) and non-classical (CD14^+^CD16^+^) monocytes were identified (**Supplementary Figure S1**). Within CD8^+^ and CD4^+^ T-cells, different subsets were gated: naïve (CD45RA^+^CD27^-^), central memory (CM, CD45RA^-^CD27^+^), effector memory (EM, CD45RA^-^CD27^-^), terminally differentiated effector memory (TEMRA, CD45RA^+^CD27^+^) and total memory (Memory) (**Supplementary Figure S2**). DCs were gated as Lin-2^-^ and HLA-DR^+^ cells, and DC subsets (myeloid, mDCs, and plasmacytoid DCs, pDCs) were gated based on CD11c and CD123 expression, respectively. mDCs subsets were gated based on CD16, CD1c and CD141 expression (**Supplementary Figure S3**).

Multiparametric flow cytometry was performed on an LRS Fortessa flow cytometer using FACS Diva software (BD Biosciences), acquiring 0.5-1×10^6^ events. Data were analyzed using the FlowJo 10.7.1 software (TreeStar). Additionally, flow cytometry data were analyzed with dimension reduction tools. Only data from CD4^+^ T-cells are shown, since no significant differences were found in the rest of the populations. Total CD4^+^ T-cells were gated as explained before (**Supplementary Figure S2**). Then, data was downsampled to 6.000 events (CD4^+^ T-cells) and concatenated. From the concatenated, unbiased hierarchical clustering was performed using Uniform Manifold Approximation and Projection (UMAP, data visualization), Phenograph (to determine the number of clusters), and FlowSOM (cluster analysis). Both concatenated and separated data in groups (Control and COVID-19) were analyzed (**Supplementary Figure S4**).

### 2.5 Tissue processing and imaging

Colon biopsies were fixed in 10% formalin, paraffin-embedded in blocks and sliced using a Leica RM2255 microtome (3 µm of thickness). Immunostainings against SARS-CoV-2 Nc and spike (S) proteins were performed as previously described with modifications (19). Samples were incubated with the primary antibodies (1:100, o/n, 4°C), and secondary antibodies (1:500, 1h, RT) (**Supplementary Table S1**), and mounted using Prolong Diamond Antifade Mountant with DAPI (Thermo Fisher Scientific). Images were taken using a Leica Stellaris 8 laser-scanning confocal microscope using the 40x air objective (HCX PL APO 40x/0.95 W.D., 0.17mm) with constant acquisition parameters. A sample from the pre-COVID period was included as a negative control of Nc and S immunolabelling. The fluorescently labeled structures were analyzed using Fiji Image J software (20). Image background was first subtracted before setting of a brightness threshold for the measurement of Nc or S markers. Images were analyzed from a maximum intensity projection. 3D reconstructions were performed with Imaris software (Bitplane).

### 2.6 EVs isolation

Total and neuronal-derived (NDEs) EVs were isolated, as previously reported (21) using the SmartSEC™ Single for EV Isolation (System Biosciences), following the manufacturer’s instructions. NDEs were obtained as previously described (16) by incubating with mouse anti-CD171 antibody in 50μL of 3% BSA (1h, RT). Samples were then incubated with 10μL of streptavidin-agarose Ultralink Resin (Thermo Fisher Scientific) in 3% of Bovine Serum Albumin (BSA) (1h, 4°C). Afterward, samples were centrifuged (800g, 10min, 4°C) and the pellet resuspended in 100μL of cold 0.05M Glycine-HCl (pH3, 5min) and centrifuged again (4000g, 10min). Supernatants were then gently mixed with 25μL of 10% BSA, 10μL of Tris-HCl (1M, pH8) and 370μL of the mammalian protein extraction reagent (M-PER, Thermo Fisher Scientific) and stored at -80°C.

### 2.7 Western blotting

EVs protein concentration was quantified using the BCA assay (BCA Protein Assay-Kit, Thermo Scientific), following the manufacturer’s instructions. EV proteins were separated using SDS-PAGE with pre-cast gels (4-20%, Bio-Rad) and transferred to nitrocellulose membranes using the TransBlot Turbo System (Bio-Rad). Blots were incubated with the primary antibody (1:500, o/n, 4°C), washed and incubated with the corresponding secondary antibody (1:5000, 2h, RT) (**Supplementary Table S1**). Blots were developed using the Pierce ECL Western Blotting Substrate (Thermo Fisher Scientific), visualized (ChemiDoc-Touch, BioRad) and analyzed by densitometry using Fiji ImageJ Software.

### 2.8 ELISA and multiplex immunoassays

Pro-inflammatory cytokines in both plasma and EVs were analyzed using the MesoScale Discovery U-Plex multiplex (IFN-γ, IL-1β, IL-6, IL-8, IL-12p70, IL-18, IP-10, MIP-1α, MIP-1β, and TNF-α) following the manufacturer’s instructions. Neurofilament (NfL) levels were analyzed in NDEs using the NEFL ELISA Human Kit (OKEH02111, Aviva Systems Biology), according to the manufacturer’s guidelines. Samples below the limit detection were considered as zero.

### 2.9 Statistics

Non-parametric statistical analyses were performed using SPSS (the Statistical Package for the Social Sciences software, SPSS v.25.0, Inc., Chicago, IL) and Prism (v.8.0, GraphPad Software, Inc.). In graphs, individual dots represent one participant (Blue: Control; Red: COVID-19). Continuous variables were expressed as medians with interquartile ranges [IQR] and categorical variables as percentages. The ROUT method was used to identify and discard outliers (Q= 0.1%). Differences between groups were analyzed by 2-tailed unpaired Mann-Whitney U test. The polyfunctionality index (P-Index) was calculated using Funky Cells software (22). P values <0.05 were considered statistically significant and are indicated in the figure legends.

## 3 Results

### 3.1 Demographic and clinical characteristics

Twenty participants who underwent COVID-19 at least 15 months before the study (15.30 [13.79-15.56]), and thirteen aged- and sex-matched Controls were enrolled. Time after SARS-CoV-2 infection ranged from 12.2 to 22.4 months. Clinical and demographical parameters are listed in **Table 1** and **Supplementary Table S2**. COVID-19 participants presented significant decreased numbers of leukocytes and neutrophils compared to Controls; no significant differences were found regarding the rest of the blood cell subsets. The percentage of participants with diabetes mellitus and hypertension was significantly higher in COVID-19 than in Control group (**Supplementary Table S2**), both comorbidities described as risk factors for COVID-19 (23). Interestingly, most of the self-reported symptoms in the COVID-19 group were associated with neurological alterations, including headache or mood manifestations (30% and 45%, respectively).

**Table 1:** Characteristics of the participants.

		Control (n=13)	COVID-19 (n=20)	p-value
Age (years)		71 [68-74]	67 [61-72]	0.12
Sex (female sex), n (%)		6 (46%)	12 (60%)	0.49
Hospitalized acute phase n (%)		N/A	9 (45%)	N/A
Erythrocytes (*10^6^/µL)		4.41 [4.09-5.10]	4.58 [4.38-5.05]	0.49
Hemoglobin (g/dL)		13.80 [12.40-15.25]	13.90 [13.03-15.23]	0.51
Hematocrit (%)		42.00 [38.00-45.00]	42.00 [40.00-45.50]	0.51
Leukocytes (*10^3^/µL)		7.81 [6.83-9.45]	6.52 [5.21-7.63]	*0.02
Lymphocytes (*10^3^/µL)		2.17 [1.79-3.11]	1.90 [1.56-2.55]	0.24
Neutrophils (*10^3^/µL)		4.59 [3.86-5.95]	3.15 [2.45-3.96]	**0.006
Lymphocytes/Neutrophils (ratio *10^3^/µL)		0.50 [0.34-0.75]	0.76 [0.38-0.89]	0.50
Monocytes (*10^3^/µL)		0.61 [0.55-0.77]	0.59 [0.39-0.68]	0.16
Eosinophils (*10^3^/µL)		0.19 [0.09-0.25]	0.11 [0.05-0.17]	0.06
Basophils (*10^3^/µL)		0.04 [0.04-0.06]	0.04 [0.02-0.06]	0.27
Lymphocytes (%)		30.00 [22.40-39.25]	37.10 [24.18-41.83]	0.50
Neutrophils (%)		59.40 [51.65-65.35]	49.60 [45.93-62.73]	0.36
Monocytes (%)		8.00 [7.25-8.75]	8.95 [7.80-9.75]	0.13
Eosinophils (%)		2.20 [1.65-3.00]	1.85 [1.15-3.02]	0.56
Basophils (%)		0.60 [0.45-0.75]	0.70 [0.50-0.92]	0.61
Self-reported symptoms after SARS-CoV-2 infection	Fatigue	N/A	7 (35%)	N/A
	Headache	N/A	6 (30%)	N/A
	Mood alterations (anxiety, depression, trouble concentrating or thinking)	N/A	9 (45%)	N/A
	Shortness of breath	N/A	4 (20%)	N/A
	Diarrhea	N/A	1 (5%)	N/A

Median and Interquartile Range [IQR]; N/A, not applicable; *p<0.05 and **p<0.01 (significant values indicated in bold).

### 3.2 Neurodegenerative and inflammatory markers in EVs

**Figure 1:**
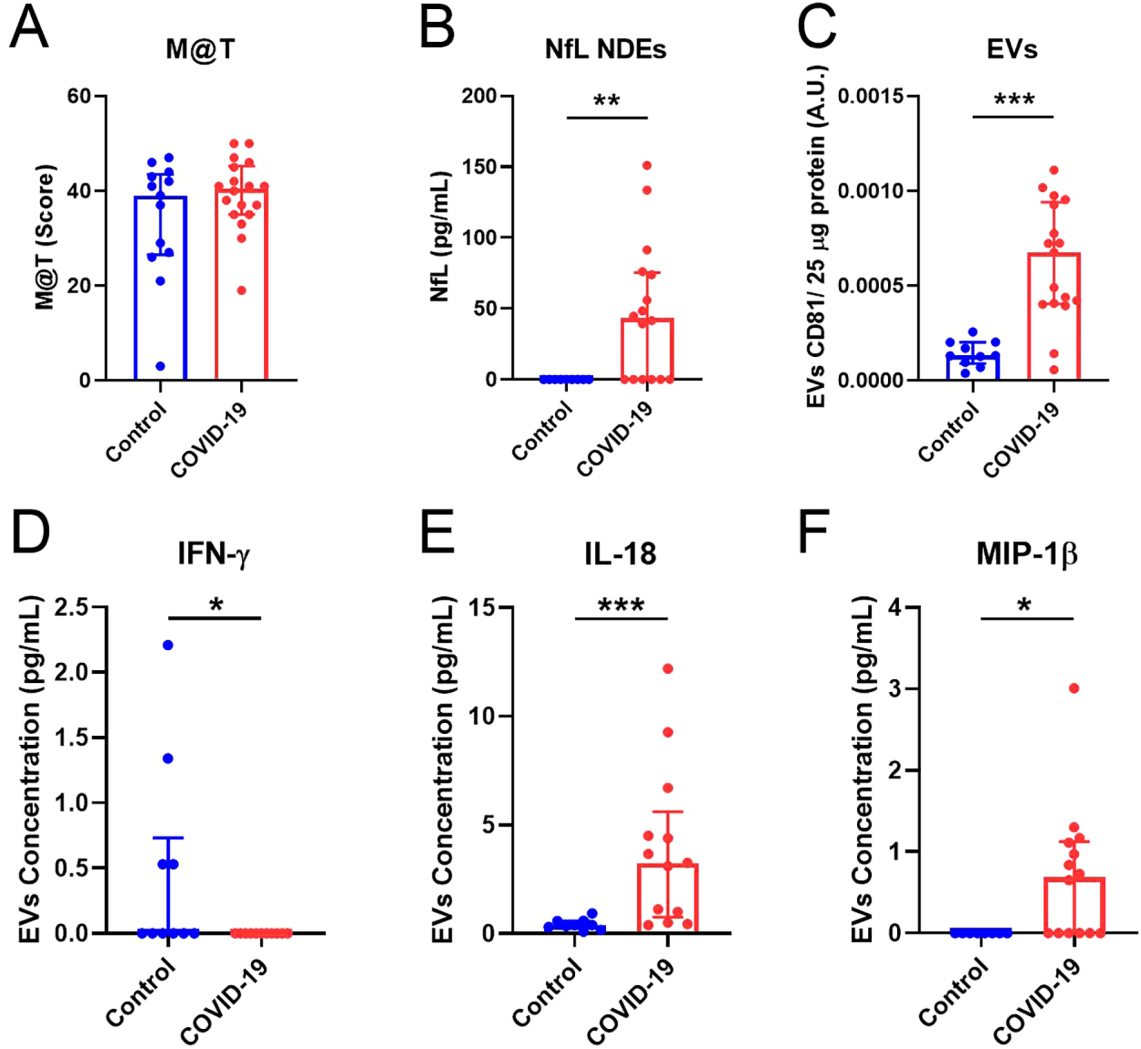
Neurodegenerative and inflammatory markers are increased in EVs of the COVID-19 group. **(A)** Total M@T score in Control and COVID-19 groups. **(B)** Quantification of NfL concentration (pg/ml) in NDEs from Control and COVID-19 participants. **(C)** Quantification of CD81 levels in EVs related to 25 μg of total protein. **(D-F)** Quantification of IFN-γ, IL-18 and MIP-1β concentration (pg/mL) in EVs, respectively. M@T, memory alteration test; NDEs, neuronal-derived extracellular vesicles; EVs: Extracellular vesicles; NfL, neurofilaments. Data are shown as median and IQR; *p<0.05, **p<0.01, ***p<0.001.

PASC involves neurological disturbances (24) and brain structure alterations (25) associated with cognitive impairment. To analyze the long-term effects of SARS-CoV-2 infection related to memory dysfunction, we used the M@T (18). Remarkably, although no differences were observed in the total M@T score (**Figure 1A**), a higher concentration of NfL was found in NDEs from COVID-19 (**Figure 1B**), suggesting that neurodegenerative processes may still be taking place 15 months after SARS-CoV-2 infection. As previously reported in acute infection (26), the levels of CD81 (a protein of the tetraspanin complex) were significantly higher in the COVID-19 group than in Controls (**Figure 1C**). Immune alterations and elevated levels of inflammatory markers have been found in COVID-19 (27). Although we did not detect differences in the concentration of pro-inflammatory cytokines in plasma (**Supplementary Figure S5**), significantly decreased concentration of IFN-γ (**Figure 1D**), but higher concentration of IL-18 (**Figure 1E**) and MIP-1β (**Figure 1F**) were detected in EVs of the COVID-19 group in comparison with Controls. No significant differences were detected for the other cytokines analyzed in EVs (**Supplementary Figure S5**). Our data suggest a higher transport of some inflammatory molecules through EVs 15 months after SARS-CoV-2 infection.

### 3.3 Monocyte immunophenotyping and function

**Figure 2:**
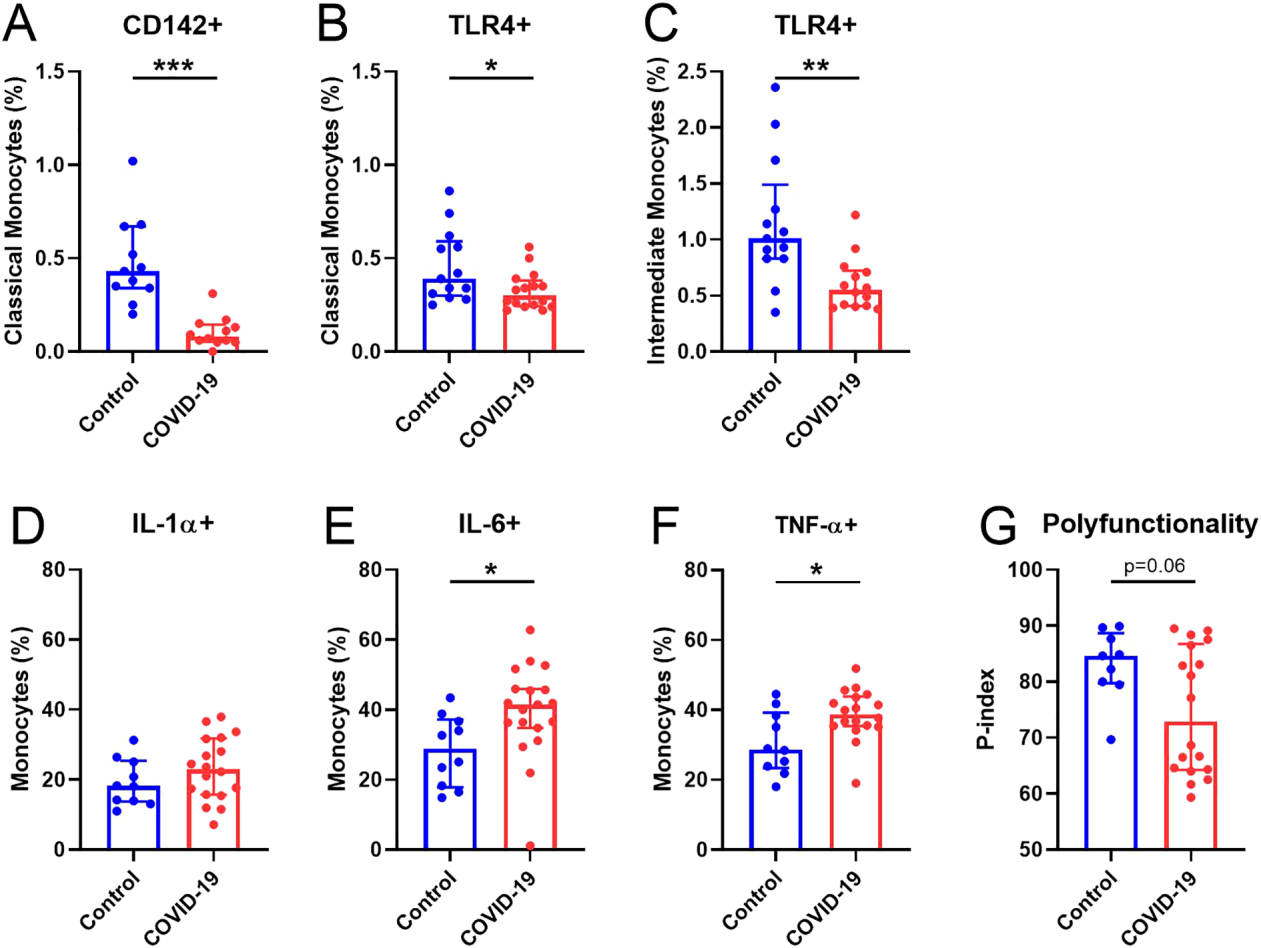
Alterations in monocytes 15 months after SARS-CoV-2 infection. **(A)** Percentage of CD142+ in classical monocytes. **(B, C)** Percentage of TLR4+ classical and intermediate monocytes, respectively. Expression of **(D)** IL-1α+, **(E)** IL-6+ and **(F)** TNF-α+ monocytes after LPS stimulation. **(G)** p-index. Data are shown as median and IQR; *p<0.05, **p<0.01, **p<0.001.

As important players in inflammation and COVID-19 severity (28), we further characterized activation and homing markers in monocytes 15 months after SARS-CoV-2 infection. Our results showed that in COVID-19 participants, there was decreased expression of classical monocytes expressing tissue factor (CD142^+^) (**Figure 2A**) and both classical and intermediate monocytes expressing the activation marker TLR4 (**Figures 2B, C**). No differences were observed in the rest of the markers analyzed, except for non-classical monocytes expressing TLR2 (**Supplementary Figure S6**). Additionally, monocytes were stimulated in a TLR4-dependent manner by adding LPS, similar to previously reported (11). Although no differences were found in the production of IL-1α (**Figure 2D**), the COVID-19 group exhibited a significantly higher production of IL-6 and TNF-α by monocytes upon LPS stimulation (**Figures 2E, F**) compared with Control group. However, the P-index tended to be lower in the COVID-19 group (**Figure 2G**), suggesting less polyfunctional monocyte response than in Control group.

### 3.4 T-cell immunophenotyping

**Figure 3:**
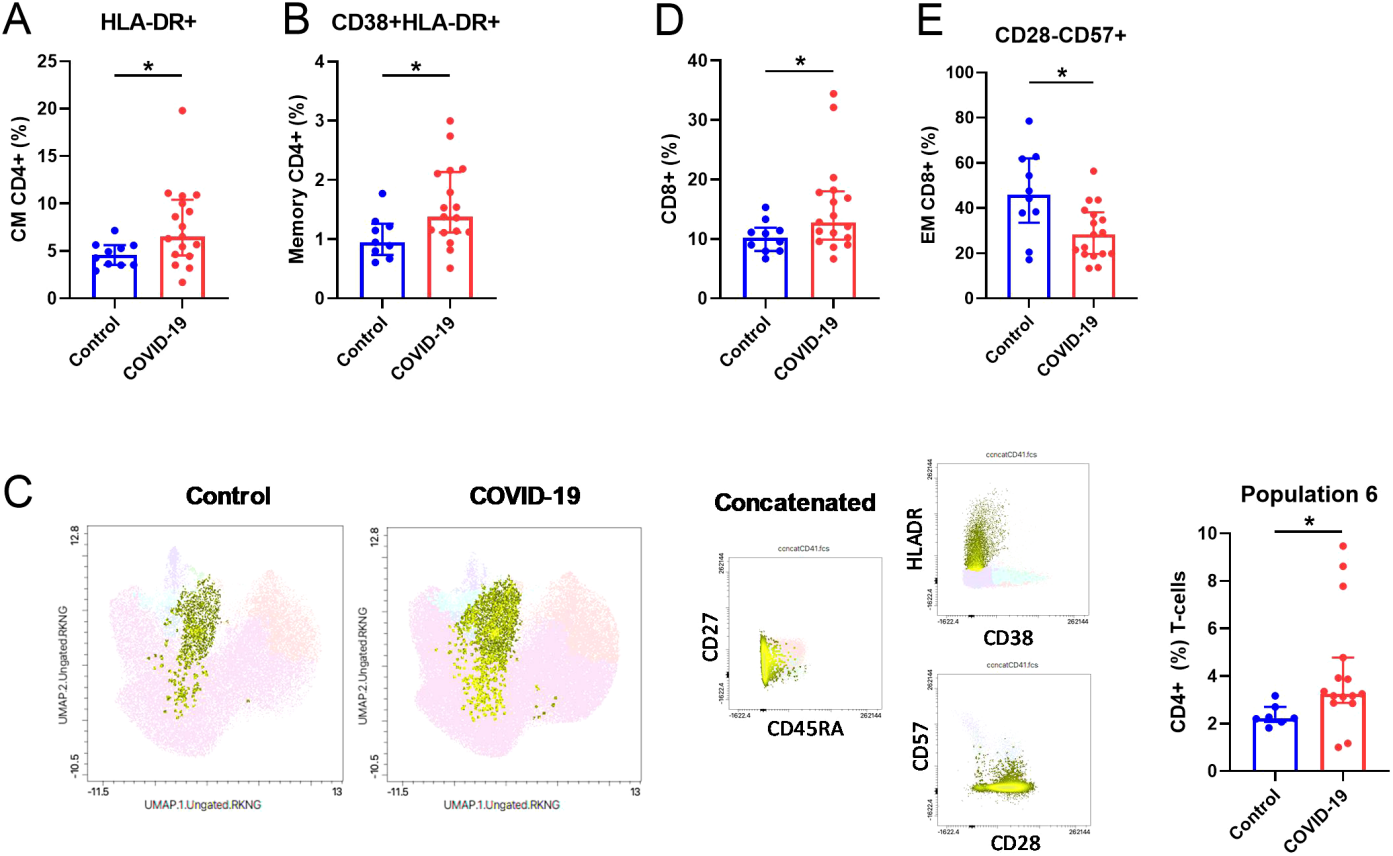
Altered T-cell parameters in COVID-19 participants compared to Controls. **(A)** Quantification of HLA-DR+ (%) CM and **(B)** CD38+HLA-DR+ Memory CD4+ T-cells. **(C)** UMAP visualization and bar graphs (right) showing the frequency of population 6 (CD45RA-HLADR++CD38-CD57-CD28+CD4+ T-cells). **(D)** Quantification of CD8+ (%) T-cells and **(E)** CD28-CD57+ in the EM CD8+ T-cell subsets. EM, effector memory; CM, central memory. Data are shown as median and IQR; *p<0.05.

We have previously reported an altered T cell pattern in previously hospitalized COVID-19 patients 7 months after SARS-CoV-2 infection (8). Fifteen months after SARS-CoV-2 infection, in CD4^+^ T-cells, we found a significant increased percentage of HLA-DR^+^ CM T-cells (**Figure 3A**) and in CD38^+^HLA-DR^+^ memory population in COVID-19 compared to Control (**Figure 3B**). In line with these results, UMAP analysis (**Figure 3C**) reported higher proportion of a specific CD4^+^ T cell population in COVID-19 (population 6, right bar graph); this population included CM and EM CD4^+^ T cells, with an activated (HLA-DR^++^) and no senescent phenotype (CD57^-^CD28^+^). A higher percentage of the bulk CD8^+^ T-cells (**Figure 3D**) was identified in the COVID-19 group. Interestingly, a lower percentage of senescent (CD28^-^CD57^+^) CD8^+^ EM T-cells were observed in COVID-19 in comparison with Control group (**Figure 3E**). No differences were found in any of the other markers analyzed for both CD4^+^ and CD8^+^ T-cells (**Supplementary Figures S7A, B**, respectively).

### 3.5 Dendritic cell immunophenotyping

**Figure 4:**
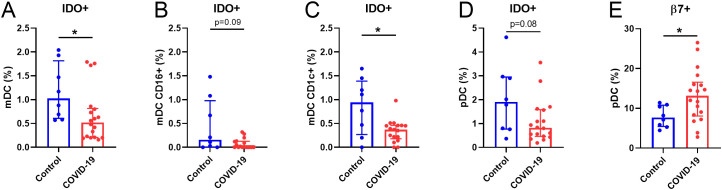
Altered DCs parameters in COVID-19 participants compared to Controls. **(A)** Quantification of IDO+ (%) in the total mDCs, **(B)** CD16+ and **(C)** CD1c+ subsets and in **(D)** pDCs populations. **(E)** Quantification of β7+ (%) in pDCs. mDCs, myeloid dendritic cells; pDCs, plasmacytoid dendritic cells. Data are shown as median and IQR; *p<0.05.

We previously reported deficiencies in DCs 7 months after SARS-CoV-2 infection (9). A deep immunophenotyping of both mDCs and pDCs revealed decreased levels of DCs expressing IDO^+^, including total mDCs (**Figure 4A**), CD16^+^ (**Figure 4B**), and CD1c^+^ (**Figure 4C**) mDCs and pDCs (**Figure 4D**) in COVID-19 compared to Control group. No differences were found in any of the other markers analyzed (**Supplementary Figure S8**). Interestingly, the percentage of pDCs expressing β7-integrin was increased in the COVID-19 group (**Figure 4E**).

### 3.6 Viral persistence in colon tissue 15 months after SARS-CoV-2 infection

**Figure 5:**
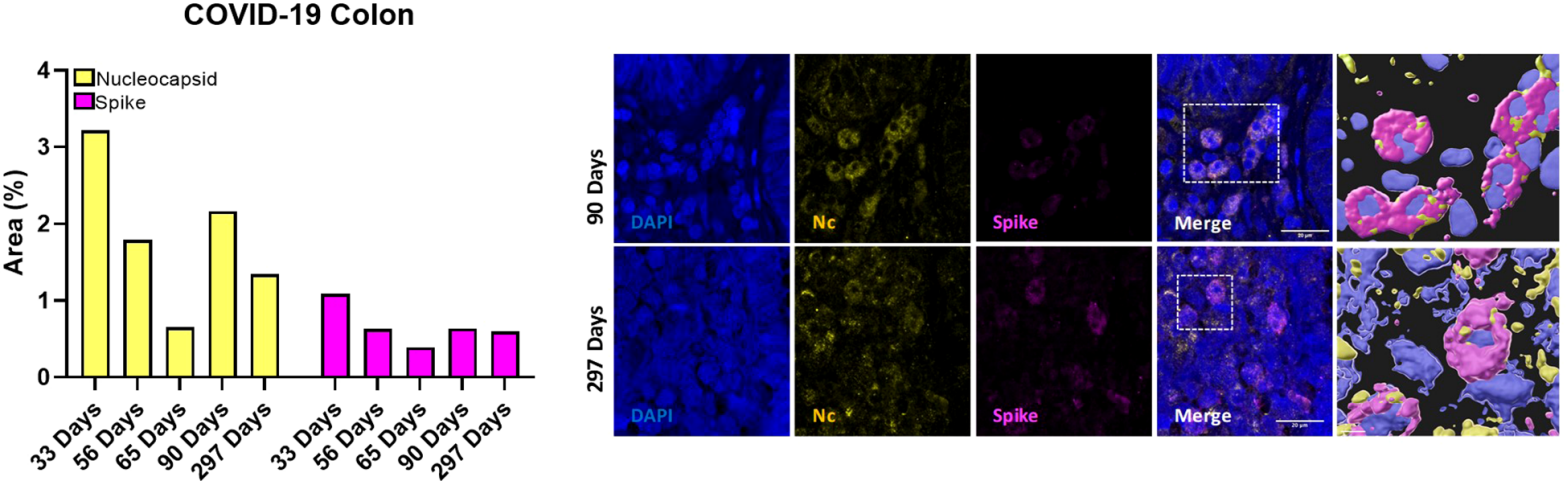
Quantification of occupied area (%) of nucleocapsid (yellow bars) and spike (magenta bars) markers in colon tissue from five participants at 33, 56, 65, 90 and 297 days after confirmed SARS-CoV-2 infection (left) and representative immunostaining images of colon biopsies from those participants (right). Blue, DAPI; yellow, nucleocapsid; magenta, spike. Scale bar: 20 μm.

pDCs expressing β7-integrin migrate from peripheral blood to colorectum tissue in simian immunodeficiency virus (SIV) infection (29). The analysis of the SARS-CoV-2 Nc and S immunolabeling in colon samples (**Figure 5**) revealed that the percentage of occupied area of both proteins decreased over the time. However, SARS-CoV-2 persistence was still observed 297 days after the infection in colon tissue.

## 4 Discussion

In this study, we aimed to determine whether immune alterations could modify EVs cargo and contribute to neurological manifestations 15 months after SARS-CoV-2 infection. First, we compared clinical parameters in Control and COVID-19 survivors. We found that leukocytes and neutrophils were significantly decreased in the COVID-19 group, suggesting a persistent immune dysregulation even in the long-term. Consistent with previous reports (30), diabetes mellitus and hypertension were the common comorbidities found in COVID-19 participants.

Other important aspects analyzed in this work were the neurological sequelae and memory alterations in COVID-19 survivors. Although both groups presented a similar cognitive M@T score, COVID-19 participants manifested cognitive complaints, referred to as “brain fog”, including a combination of memory alterations, fatigue, and lack of motivation (31). Interestingly, COVID-19 participants still had higher levels of NfL in NDEs, as previously reported in COVID-19 survivors 1-3 months after the infection (17). These findings may indicate that individuals who recovered from COVID-19 present neuronal injury associated with the disease (25), suggesting a potential link between viral infection and long-term neurological sequelae (24).

Increased peripheral inflammatory mediators associated with cognitive impairment have been described in hospitalized severe COVID-19 survivors after 12 months (32). IL-18 has been associated with cardiopulmonary inflammation in acute SARS-CoV-2 infection (33), and elevated levels of MIP-1β and IFN-γ have been found in the plasma of long-COVID individuals with symptoms (34, 35). Moreover, higher levels of CD81 EVs colocalizing with SARS-CoV-2 and pro-inflammatory cytokines have been found in sputum (15) and plasma (36) samples in acute infection. Despite the lack of differences in plasma cytokines, we detected higher concentrations of IL-18 and MIP-1β as well as CD81 in EVs from COVID-19 participants, suggesting that they may play a fundamental role in the altered immune response 15 months after the acute infection. Interestingly, higher levels of NfL were found in NDEs of recovered COVID-19 patients between one and three months after acute infection (17). Although we did not detect memory alterations, the higher levels of NfL in NDEs may indicate neuronal injury in COVID-19 participants that could lead to cognitive dysfunction in the future or that our study was underpowered to detect changes in memory.

Related to the immune system, previous works from our laboratory have demonstrated dysregulation of innate immune cells in the elderly, associated with cognitive decline (37, 38) and SARS-CoV-2 mRNA vaccine response (11), as well as 7 months after acute SARS-CoV-2 infection (8, 9). In this study, the deep-immunophenotyping of monocytes, T-cells, and DCs unveiled notable differences between Control and COVID-19 participants. Decreased expression of tissue factor (CD142^+^) and activation markers (TLR4^+^) in monocytes were found in COVID-19. Diminished monocyte markers have been reported in severe disease (39) and convalescent COVID-19 survivors (40), which point toward persistent dysregulation of innate immune responses. We found that the percentage of monocytes producing IL-6 and TNF-α upon LPS stimulation was increased in the COVID-19 group, while a lower polyfunctionality response was observed, suggesting that monocytes of these patients are capable of producing more inflammatory mediators but showed a lower response quality. In contrast, monocytes from convalescent COVID-19 (2-4 weeks after disease onset) produced less TNF-α and IL-6 in response to LPS stimulation (40), which may indicate a time-dependent modification of the monocyte response after infection. Although with fewer differences than the reported 7 months after acute infection (8), the increased expression of activation markers in effector CD4^+^ T-cells found in the COVID-19 group suggests an ongoing immune activation that may be participating in PASC, which might also be related to the higher pro-inflammatory and less polyfunctional monocyte pattern observed in these participants. Moreover, diabetes and hypertension might also be other factors promoting this persistent immune activation/inflammation (41) in the COVID-19 group; these factors that have been related with higher risk of suffering long-term cardiovascular diseases (42). We also found diminished expression of IDO on several DC subsets in COVID-19, as previously reported by us seven months after the acute infection (9). IDO is an enzyme produced by DCs among others, with a key role in immune tolerance (43). A possible viral persistence along the time may cause the exhaustion of DCs producing IDO and/or migration of these cells to inflammatory focus. Additionally, due to the involvement of IDO-expressing cells in T cell response suppression, lower percentages of these IDO^+^ DCs might partially explain the higher T cell activation found in the COVID-19 group. We also observed a higher percentage of pDCs expressing β7-integrin in COVID-19. Although a lower percentage of pDCs expressing β7-integrin in peripheral blood was found seven months after acute SARS-CoV-2 infection (9), the upregulation of β7^+^ pDCs has been associated with their rapid recruitment to the colorectum tissue after a pathogenic SIV infection (29). In line with this finding, SARS-CoV-2 was still present 297 days after acute infection, indicating a possible connection between gut and peripheral blood immunity and viral persistence. This persistence could potentially explain the elevated monocyte-mediated inflammation, increased T-cell activation, and the potential migration of DCs observed in the COVID-19 group.

One of the major limitations of our study was the low number of participants included; however, significant differences were found between groups and the differences were reproducible among several cell subjects. Furthermore, we conducted a deep-immunophenotyping of different immune cells and pro-inflammatory cytokines in both plasma and EVs samples. Unfortunately, five out of eighteen Controls initially included were in the acute phase of infection at the time of blood sampling and had to be discarded from the final analysis. Another limitation was that the last time point of colon biopsies was collected at 297 days, which may not be sufficient to fully elucidate the long-term effects of SARS-CoV-2 infection in colon tissue. Further studies with longer follow-up periods are needed to better understand post-acute sequelae of SARS-CoV-2 infection.

Altogether, our results reveal persistent immune and neurodegenerative- related alterations 15 months after SARS-CoV-2 infection. These findings underline the importance of understanding the mechanisms behind the immune deficits associated with the long-term consequences of PASC. Further studies are needed to develop targeted interventions for the management of PASC. Additionally, our results demonstrated the potential of EVs as a promising tool for the early identification of neuronal injury. Combined with the measurement of immunological alterations, this approach may aid in diagnosing virus-induced neurological disorders.

## Data Availability

The original contributions presented in the study are included in the article/**Supplementary Material**. Further inquiries can be directed to the corresponding authors.

## References

[1] Wu F Zhao S Yu B Chen YM Wang W Song ZG. A new coronavirus associated with human respiratory disease in China. Nature. (2020) 579(7798):265–9. doi: 10.1038/s41586-020-2008-3 PMC7094943 32015508

[2] WHO. World Health Organization. WHO coronavirus disease (COVID-19) dashboard with vaccination data (2023). Available online at: https://covid19.who.int/ (Accessed December, 2023).

[3] Agrawal U Bedston S McCowan C Oke J Patterson L Robertson C. Severe COVID-19 outcomes after full vaccination of primary schedule and initial boosters: pooled analysis of national prospective cohort studies of 30 million individuals in England, Northern Ireland, Scotland, and Wales. Lancet. (2022) 400(10360):1305–20. doi: 10.1016/S0140-6736(22)01656-7 PMC9560746 36244382

[4] Hastie CE Lowe DJ McAuley A Mills NL Winter AJ Black C. True prevalence of long-COVID in a nationwide, population cohort study. Nat Commun. (2023) 14(1):7892. doi: 10.1038/s41467-023-43661-w 38036541 PMC10689486

[5] Espin E Yang C Shannon CP Assadian S He D Tebbutt SJ. Cellular and molecular biomarkers of long COVID: a scoping review. EBioMedicine. (2023) 91:104552. doi: 10.1016/j.ebiom.2023.104552 37037165 PMC10082390

[6] Ahmed H Patel K Greenwood DC Halpin S Lewthwaite P Salawu A. Long-term clinical outcomes in survivors of severe acute respiratory syndrome and Middle East respiratory syndrome coronavirus outbreaks after hospitalisation or ICU admission: A systematic review and meta-analysis. J Rehabil Med. (2020) 52(5):jrm00063. doi: 10.2340/16501977-2694 32449782

[7] Prudencio M Erben Y Marquez CP Jansen-West KR Franco-Mesa C Heckman MG. Serum neurofilament light protein correlates with unfavorable clinical outcomes in hospitalized patients with COVID-19. Sci Transl Med. (2021) 13(602):eabi7643. doi: 10.1126/scitranslmed.abi7643 34131052 PMC8432951

[8] Perez-Gomez A Gasca-Capote C Vitalle J Ostos FJ Serna-Gallego A Trujillo-Rodriguez M. Deciphering the quality of SARS-CoV-2 specific T-cell response associated with disease severity, immune memory and heterologous response. Clin Transl Med. (2022) 12(4):e802. doi: 10.1002/ctm2.802 35415890 PMC9005926

[9] Perez-Gomez A Vitalle J Gasca-Capote C Gutierrez-Valencia A Trujillo-Rodriguez M Serna-Gallego A. Dendritic cell deficiencies persist seven months after SARS-CoV-2 infection. Cell Mol Immunol. (2021) 18(9):2128–39. doi: 10.1038/s41423-021-00728-2 PMC8294321 34290398

[10] Dan JM Mateus J Kato Y Hastie KM Yu ED Faliti CE. Immunological memory to SARS-CoV-2 assessed for up to 8 months after infection. Science. (2021) 371(6529):eabf4063. doi: 10.1126/science.abf4063 33408181 PMC7919858

[11] Vitalle J Perez-Gomez A Ostos FJ Gasca-Capote C Jimenez-Leon MR Bachiller S. Immune defects associated with lower SARS-CoV-2 BNT162b2 mRNA vaccine response in aged people. JCI Insight. (2022) 7(17):e161045. doi: 10.1172/jci.insight.161045 35943812 PMC9536264

[12] Collier DA Ferreira I Kotagiri P Datir RP Lim EY Touizer E. Age-related immune response heterogeneity to SARS-CoV-2 vaccine BNT162b2. Nature. (2021) 596(7872):417–22. doi: 10.1038/s41586-021-03739-1 PMC8373615 34192737

[13] Muller L Andree M Moskorz W Drexler I Walotka L Grothmann R. Age-dependent immune response to the Biontech/Pfizer BNT162b2 coronavirus disease 2019 vaccination. Clin Infect Dis. (2021) 73(11):2065–72. doi: 10.1093/cid/ciab381 PMC8135422 33906236

[14] Pulliam L Gupta A. Modulation of cellular function through immune-activated exosomes. DNA Cell Biol. (2015) 34(7):459–63. doi: 10.1089/dna.2015.2884 PMC4504252 25945690

[15] Sun R Cai Y Zhou Y Bai G Zhu A Kong P. Proteomic profiling of single extracellular vesicles reveals colocalization of SARS-CoV-2 with a CD81/integrin-rich EV subpopulation in sputum from COVID-19 severe patients. Front Immunol. (2023) 14:1052141. doi: 10.3389/fimmu.2023.1052141 37251406 PMC10214957

[16] Barberis E Vanella VV Falasca M Caneapero V Cappellano G Raineri D. Circulating exosomes are strongly involved in SARS-CoV-2 infection. Front Mol Biosci. (2021) 8:632290. doi: 10.3389/fmolb.2021.632290 33693030 PMC7937875

[17] Sun B Tang N Peluso MJ Iyer NS Torres L Donatelli JL. Characterization and biomarker analyses of post-COVID-19 complications and neurological manifestations. Cells. (2021) 10(2):386. doi: 10.3390/cells10020386 33668514 PMC7918597

[18] Rami L Molinuevo JL Sanchez-Valle R Bosch B Villar A. Screening for amnestic mild cognitive impairment and early Alzheimer’s disease with M@T (Memory Alteration Test) in the primary care population. Int J Geriatr Psychiatry. (2007) 22(4):294–304. doi: 10.1002/gps.v22:4 16998781

[19] Bachiller S Hidalgo I Garcia MG Boza-Serrano A Paulus A Denis Q. Early-life stress elicits peripheral and brain immune activation differently in wild type and 5xFAD mice in a sex-specific manner. J Neuroinflammation. (2022) 19(1):151. doi: 10.1186/s12974-022-02515-w 35705972 PMC9199174

[20] Schindelin J Arganda-Carreras I Frise E Kaynig V Longair M Pietzsch T. Fiji: an open-source platform for biological-image analysis. Nat Methods. (2012) 9(7):676–82. doi: 10.1038/nmeth.2019 PMC3855844 22743772

[21] Peluso MJ Deeks SG Mustapic M Kapogiannis D Henrich TJ Lu S. SARS-CoV-2 and mitochondrial proteins in neural-derived exosomes of COVID-19. Ann Neurol. (2022) 91(6):772–81. doi: 10.1002/ana.26350 PMC9082480 35285072

[22] Larsen M Sauce D Arnaud L Fastenackels S Appay V Gorochov G. Evaluating cellular polyfunctionality with a novel polyfunctionality index. PloS One. (2012) 7(7):e42403. doi: 10.1371/journal.pone.0042403 22860124 PMC3408490

[23] Yang X Yu Y Xu J Shu H Xia J Liu H. Clinical course and outcomes of critically ill patients with SARS-CoV-2 pneumonia in Wuhan, China: a single-centered, retrospective, observational study. Lancet Respir Med. (2020) 8(5):475–81. doi: 10.1016/S2213-2600(20)30079-5 PMC7102538 32105632

[24] Alonso-Bellido IM Bachiller S Vazquez G Cruz-Hernandez L Martinez E Ruiz-Mateos E. The other side of SARS-CoV-2 infection: Neurological sequelae in patients. Front Aging Neurosci. (2021) 13:632673. doi: 10.3389/fnagi.2021.632673 33889082 PMC8055831

[25] Douaud G Lee S Alfaro-Almagro F Arthofer C Wang C McCarthy P. SARS-CoV-2 is associated with changes in brain structure in UK biobank. Nature. (2022) 604(7907):697–707. doi: 10.1101/2021.06.11.21258690 35255491 PMC9046077

[26] Balbi C Burrello J Bolis S Lazzarini E Biemmi V Pianezzi E. Circulating extracellular vesicles are endowed with enhanced procoagulant activity in SARS-CoV-2 infection. EBioMedicine. (2021) 67:103369. doi: 10.1016/j.ebiom.2021.103369 33971404 PMC8104913

[27] Lyra ESNM Barros-Aragao FGQ De Felice FG Ferreira ST. Inflammation at the crossroads of COVID-19, cognitive deficits and depression. Neuropharmacology. (2022) 209:109023. doi: 10.1016/j.neuropharm.2022.109023 35257690 PMC8894741

[28] Trujillo-Rodriguez M Munoz-Muela E Serna-Gallego A Praena-Fernandez JM Perez-Gomez A Gasca-Capote C. Clinical, laboratory data and inflammatory biomarkers at baseline as early discharge predictors in hospitalized SARS-CoV-2 infected patients. PloS One. (2022) 17(7):e0269875. doi: 10.1371/journal.pone.0269875 35834501 PMC9282584

[29] Kwa S Kannanganat S Nigam P Siddiqui M Shetty RD Armstrong W. Plasmacytoid dendritic cells are recruited to the colorectum and contribute to immune activation during pathogenic SIV infection in rhesus macaques. Blood. (2011) 118(10):2763–73. doi: 10.1182/blood-2011-02-339515 PMC3172794 21693759

[30] Zhou F Yu T Du R Fan G Liu Y Liu Z. Clinical course and risk factors for mortality of adult inpatients with COVID-19 in Wuhan, China: a retrospective cohort study. Lancet. (2020) 395(10229):1054–62. doi: 10.1016/S0140-6736(20)30566-3 PMC7270627 32171076

[31] Shimohata T. Neuro-COVID-19. Clin Exp Neuroimmunol. (2022) 13(1):17–23. doi: 10.1111/cen3.12676 34899999 PMC8652810

[32] Group P-CC. Clinical characteristics with inflammation profiling of long Covid and association with 1-year recovery following hospitalisation in the UK: a prospective observational study. Lancet Respir Med. (2022) 10(8):761–75. doi: 10.1016/S2213-2600(22)00127-8 PMC9034855 35472304

[33] Liang S Bao C Yang Z Liu S Sun Y Cao W. SARS-CoV-2 spike protein induces IL-18-mediated cardiopulmonary inflammation *via* reduced mitophagy. Signal Transduct Target Ther. (2023) 8(1):108. doi: 10.1038/s41392-023-01368-w 36894537 PMC9998025

[34] Dhingra S Fu J Cloherty G Mallon P Wasse H Moy J. Identification of inflammatory clusters in long-COVID through analysis of plasma biomarker levels. Front Immunol. (2024) 15:1385858. doi: 10.3389/fimmu.2024.1385858 38745674 PMC11091280

[35] Krishna BA Lim EY Metaxaki M Jackson S Mactavous L BioResource N. Spontaneous, persistent, T cell-dependent IFN-gamma release in patients who progress to Long Covid. Sci Adv. (2024) 10(8):eadi9379. doi: 10.1126/sciadv.adi9379 38381822 PMC10881041

[36] Chen L Chen R Yao M Feng Z Yuan G Ye F. COVID-19 plasma exosomes promote proinflammatory immune responses in peripheral blood mononuclear cells. Sci Rep. (2022) 12(1):21779. doi: 10.1038/s41598-022-26457-8 36526691 PMC9756928

[37] de Pablo-Bernal RS Canizares J Rosado I Galva MI Alvarez-Rios AI Carrillo-Vico A. Monocyte phenotype and polyfunctionality are associated with elevated soluble inflammatory markers, cytomegalovirus infection, and functional and cognitive decline in elderly adults. J Gerontol A Biol Sci Med Sci. (2016) 71(5):610–8. doi: 10.1093/gerona/glv121 PMC5007736 26286603

[38] Ferrando-Martinez S Ruiz-Mateos E Casazza JP de Pablo-Bernal RS Dominguez-Molina B Munoz-Fernandez MA. IFNgamma(-)TNFalpha(-)IL2(-)MIP1alpha(-)CD107a(+)PRF1(+) CD8 pp65-specific T-cell response is independently associated with time to death in elderly humans. J Gerontol A Biol Sci Med Sci. (2015) 70(10):1210–8. doi: 10.1093/gerona/glu171 PMC4612356 25238774

[39] Paliogiannis P Zinellu A Scano V Mulas G De Riu G Pascale RM. Laboratory test alterations in patients with COVID-19 and non COVID-19 interstitial pneumonia: a preliminary report. J Infect Dev Ctries. (2020) 14(7):685–90. doi: 10.3855/jidc.12879 32794454

[40] Ravkov EV Williams E Elgort M Barker AP Planelles V Spivak AM. Reduced monocyte proportions and responsiveness in convalescent COVID-19 patients. Front Immunol. (2023) 14:1329026. doi: 10.3389/fimmu.2023.1329026 38250080 PMC10797708

[41] Petrie JR Guzik TJ Touyz RM. Diabetes, hypertension, and cardiovascular disease: Clinical insights and vascular mechanisms. Can J Cardiol. (2018) 34(5):575–84. doi: 10.1016/j.cjca.2017.12.005 PMC5953551 29459239

[42] Savedchuk S Raslan R Nystrom S Sparks MA. Emerging viral infections and the potential impact on hypertension, cardiovascular disease, and kidney disease. Circ Res. (2022) 130(10):1618–41. doi: 10.1161/CIRCRESAHA.122.320873 35549373

[43] Mellor AL Munn DH. IDO expression by dendritic cells: tolerance and tryptophan catabolism. Nat Rev Immunol. (2004) 4(10):762–74. doi: 10.1038/nri1457 15459668

