# Supplementary material for: SARS-CoV-2 post-acute sequelae linked to inflammation via extracellular vesicles
Source: Front Immunol. 2025 Apr 16;16:1501666. doi: 10.3389/fimmu.2025.1501666 (PMC12052859; doi:10.3389/fimmu.2025.1501666)
Supplement: Supplementary file 1 [file DataSheet1.pdf]

Supplementary Material

1 Supplementary Figures and Tables

1.1 Supplementary Table 1. List of antibodies.

EXPERIMENT	NAME	FLUOROCHROME	CLONE	SOURCE
Monocytes phenotyping	Mouse anti-human CD3	V450	SP34-2	BD Biosciences
	Mouse anti-human CD19	V450	HIB19	
	Mouse anti-human CD20	V450	L27	
	Mouse anti-human CD56	V450	B159	
	Mouse anti-human CD14	BV650	M5E2	
	Mouse anti-human CD16	PeCF594	3G8	
	Mouse anti-human TLR4	BV786	TF901	
	Mouse anti-human CD142	Pe	HTF-1	
	Mouse anti-human CCR5	APC-Cy7	2D7/CCR5	
	Mouse anti-human HLA-DR	BV570	L243	Biolegend
	Mouse anti-human TLR2	FITC	TL2.1	
	Mouse anti-human CD40	APC	HB14	
	Mouse anti-human CX3CR1	PerCP-Cy5.5	2A9-1	
	Mouse anti-human CCR2	BV605	K036C2	
	Mouse anti-human CD49d	BV711	9F10	
	Mouse anti-human CD11b	AF700	VIM12	Thermo Fisher Scientific
Monocytes functionality	Mouse anti-human IL-6	Pe	MQ2-6A3	BD Biosciences
	Mouse anti-human IL-1α	FITC	AS5	

	Mouse anti-human TNF-α	AF700	MAb11	
T-cells phenotyping	Mouse anti-human CD8	PerCP-Cy5.5	SK1	Biolegend
	Mouse anti-human CD45RA	PeCy7	L48	
	Mouse anti-human CD3	BV711	SP34-2	
	Mouse anti-human HLA-DR	BV570	L243	
	Mouse anti-human CD161	BV421	HP-3G10	
	Mouse anti-human ki67	FITC	11F6	
DCs phenotyping	Mouse anti-human CD11c	BV650	B-ly6	BD Biosciences
	Mouse anti-human HLA-DR	BV711	G46-6	
	Mouse anti-human CD16	BV605	3G8	
	Mouse anti-human CCR7 (CD197)	BV786	3D12	
	Mouse anti-human CD86	BV421	2331 (FUN-1)	
	Mouse anti-human PD-L1 (CD197)	PeCF594	MIH1	
	Mouse anti-human Integrin- β7	APC	FIB504	
	Mouse anti-human CD4	PerCP-Cy5.5	OKT4	Biolegend
	Mouse anti-human CD1c	APC-Cy7	L161	
	Mouse anti-human CD141	PeCy7	M80	
	Mouse anti-human CD123	AF700	32703	R&D Systems
	Mouse anti-human IDO	Pe	eyedio	eBioscience
Immunostainings	Rabbit-anti-SARS-CoV-2 Nucleocapsid	N/A	N/A	Novus Biologicals
	Mouse-anti-SARS-CoV-2 Spike	N/A	1A9	GeneTex
	Donkey-anti-rabbit	AF555	N/A	Invitrogen
	Donkey-anti-mouse	AF647	N/A	

NDEs Isolation	Mouse anti-human L1CAM	N/A	5G3	eBiosciences
EVs Western Blots	Mouse anti-human CD81	N/A	M38	Thermo Fisher Scientific
	Peroxidase-conjugated anti-Ig	N/A	N/A	DAKO

N/A: Not applicable.

1.2 Supplementary Table 2. Main comorbidities in Control and COVID-19 groups.

	HD (n=13)	COVID-19 (n=20)	p-value
Diabetes mellitus	1 (7%)	7 (35%)	***0.04**
Hypertension	1 (7%)	7 (35%)	***0.04**
Cardiovascular Disease	3 (23%)	3 (15%)	>0.99
Arthrosis	1 (7%)	0 (0%)	0.47
Obstructive pulmonary disease	2 (15%)	0 (0%)	0.21
Crohn’s disease	0 (0%)	1 (5%)	>0.99
Cancer	0 (0%)	1 (5%)	>0.99
Kidney Transplant	0 (0%)	1 (5%)	>0.99
Essential tremor	1 (7%)	1 (5%)	>0.99

Number of participants with at least one comorbidity and percentage (%); All comorbidities were diagnosed before SARS-CoV-2 infection; *p<0.05.

1.3 **Supplementary Figure 1. Gating strategy of monocytes.** Cells were firstly gated according to the size (FSC-A) and complexity (SSC-A) and Dump channel negative cells were selected (Dump channel: viability marker, CD56, CD3, CD19, CD20). Then, for monocyte identification, HLA-DR+ cells were selected and classical (CD14++CD16-), intermediate (CD14++CD16+) and non-classical (CD14+CD16+) monocytes were identified.

1.4 **1.4 Supplementary Figure 2. Gating strategy of T-cells.** Cells were firstly gated according to the size (FSC-A) and complexity (SSC-A) and viable cells were selected (viability marker negative cells). CD4 and CD8 T cells were identified as CD8-CD3+ and CD8+CD3+ cells, respectively. Then, within CD8+ and CD4+ T-cells, the following subsets were gated: naïve (CD45RA+CD27-), central memory (CM, CD45RA-CD27+), effector memory (EM, CD45RA-CD27-), terminally differentiated effector memory (TEMRA, CD45RA+CD27+) and total memory (Memory).**Supplementary Figure 3. Gating strategy of Dendritic cells.** Cells were firstly gated according to the size (FSC-A) and complexity (SSC-A) and viable cells were selected (viability marker negative cells). DCs were gated as Lineage-2- (Lin-2: CD3, CD14, CD56, CD20, CD19) and HLA-DR+ cells, and DC subsets, myeloid (mDCs) and plasmacytoid dendritic cells (pDCs), were gated based on CD11c and CD123 expression, respectively. mDCs subsets were gated based on CD16, CD1c and CD141 expression.

1.5 **Supplementary Figure 4. UMAP Visualization of total CD4+ T-cell clusters.** A) UMAPs and (B) bar graphs representing CD4+ T-cell clusters identified by FlowSOM analysis based on the expression of activation, proliferation and senescence markers in all participants. C)

Heatmap of relative expression of activation, proliferation and senescence CD4+ T-cell markers. Concatenated data including both Control and COVID-19 groups is shown (n=23).

1.6 Supplementary Figure 5. Quantification of pro-inflammatory cytokines. No differences in the concentration of plasma and EVs pro-inflammatory cytokines between Control and COVID-19 groups. D) Quantification of pro-inflammatory cytokines (IFN-γ, IL-12, IL18, IL-1β, IL-6, IP-10, MIP-1α, MIP-1β and TNF-α) concentration (pg/mL) in plasma and EVs. EVs, extracellular vesicles. Data are shown as median and IQR.

1.7 Supplementary Figure 6. Immunophenotyping of classical, intermediate and non-classical monocytes. For each subset, percentage of the following markers were analyzed: total subset, CCR2+, CCR5+, CD11b+, CD40+, CD49d+, CD142+, CX3CR1+, TLR2+ and TLR4. Data are shown as median and IQR.

1.8 Supplementary Figure 7. Immunophenotyping of T-cell subsets. Immunophenotyping of A) CD4+ and B) CD8+ T-Cell subsets. For each cell type and subset, we analyzed the following markers: total CD4+ or CD8+ cells (%), CD28-CD57+ (%), CD38+ (%), CD38+HLA-DR+ (%), CD161+ (%), HLA-DR+ (%), Ki67+ (%). CM, central memory; EM, effector memory. Data are shown as median and IQR.

1.9 Supplementary Figure 8. Immunophenotyping of Dendritic cells. Within each subset (total mDCs, CD16+, CD141+, CD1c+ and pDCs), the following markers were analyzed: β7+ (%), CD4+ (%), CD86+ (%), IDO+ (%), PDL1+ (%). mDC: myeloid Dendritic Cells; pDC: plasmacytoid Dendritic Cells. Data are shown as median and IQR.
