# Supplementary figures and images for: SARS-CoV-2 post-acute sequelae linked to inflammation via extracellular vesicles
Source: Front Immunol. 2025 Apr 16;16:1501666. doi: 10.3389/fimmu.2025.1501666 (PMC12052859; doi:10.3389/fimmu.2025.1501666)

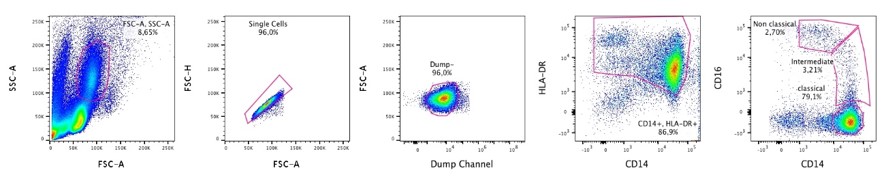

Supplement: Supplementary file 2 [file Image1.jpeg]

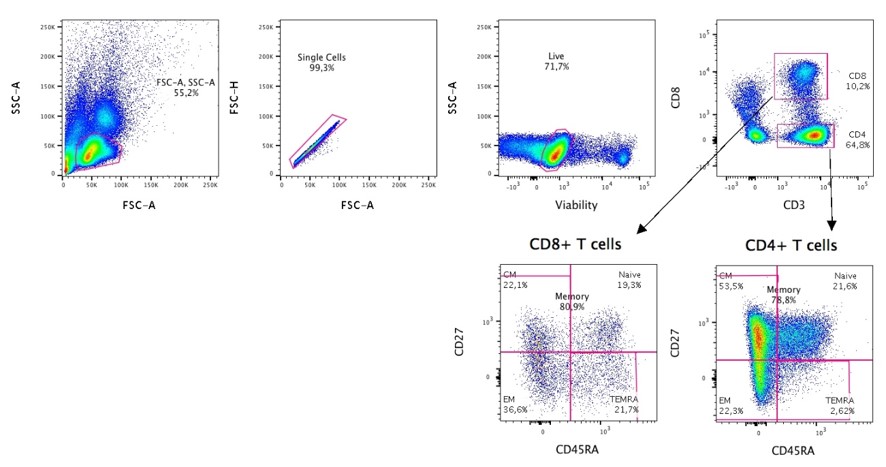

Supplement: Supplementary file 3 [file Image2.jpeg]

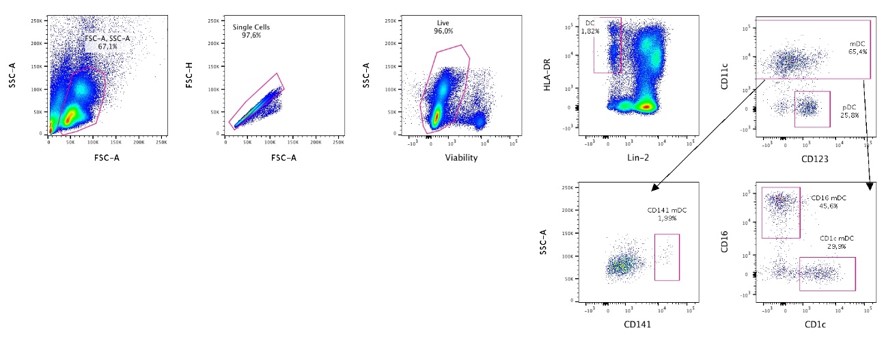

Supplement: Supplementary file 4 [file Image3.jpeg]

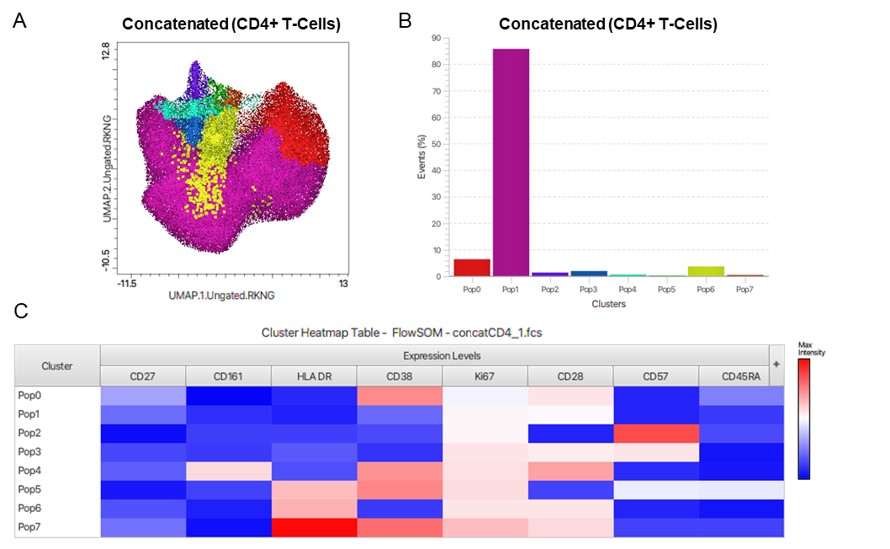

Supplement: Supplementary file 5 [file Image4.jpeg]

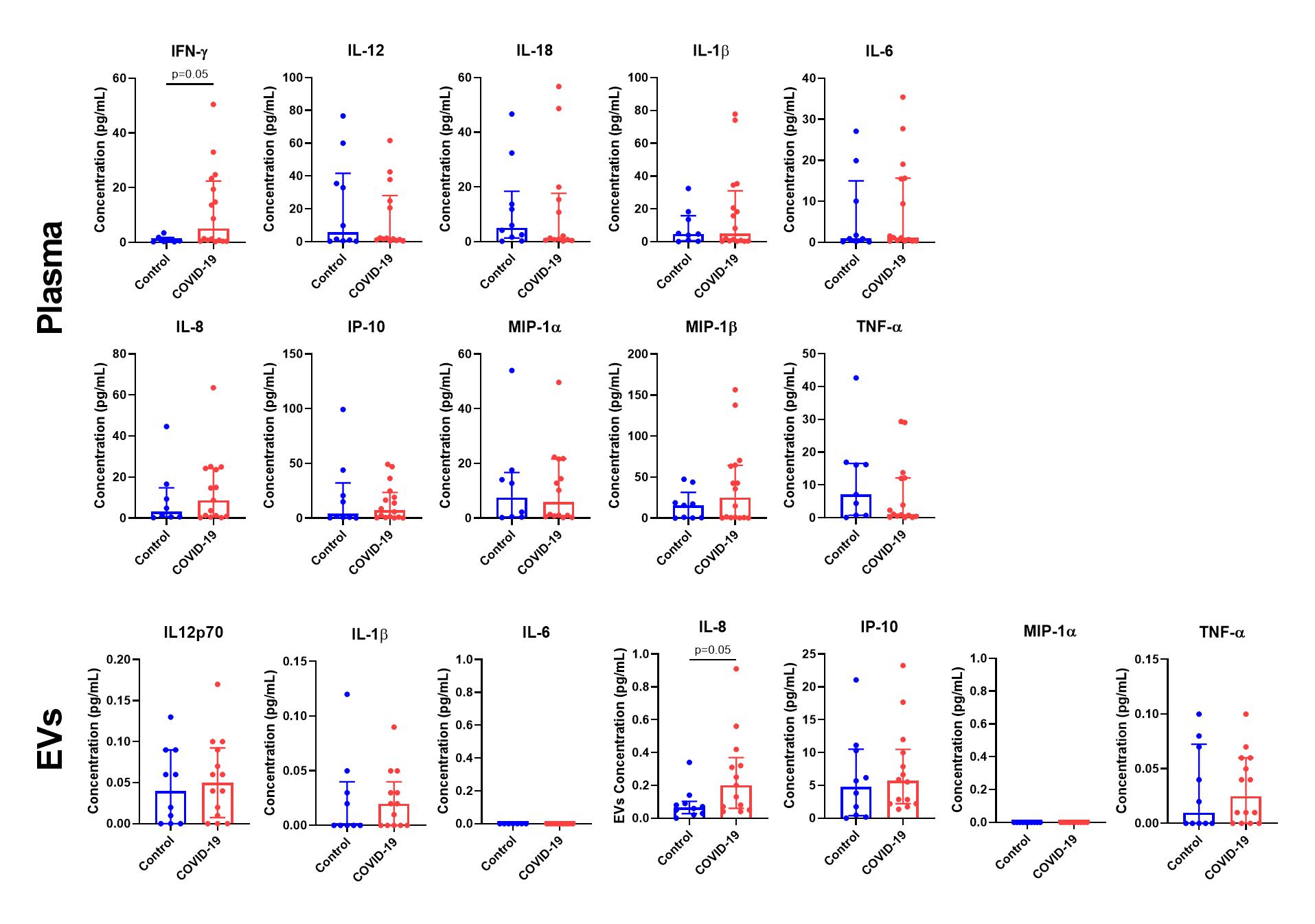

Supplement: Supplementary file 6 [file Image5.jpeg]

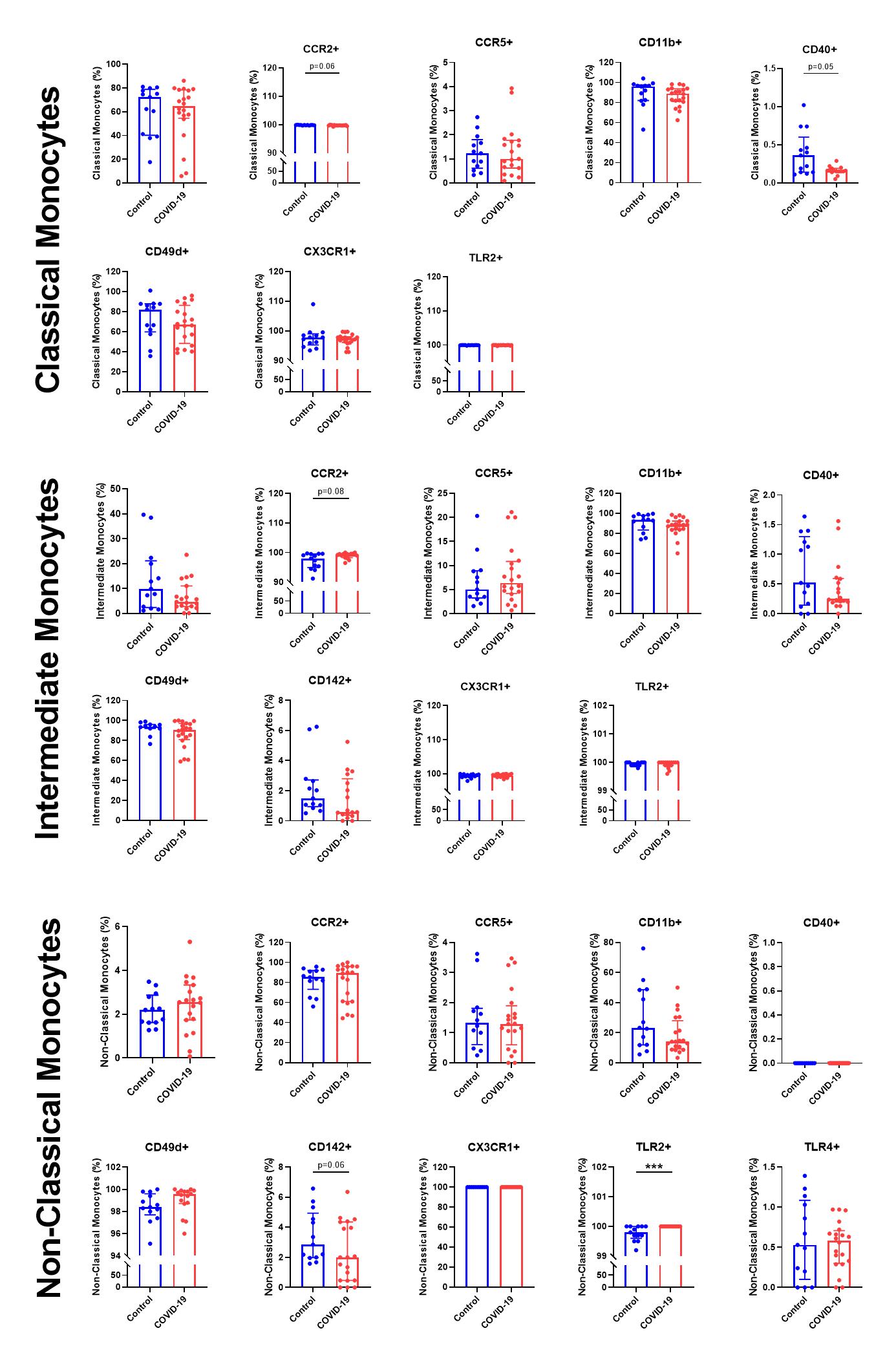

Supplement: Supplementary file 7 [file Image6.jpeg]

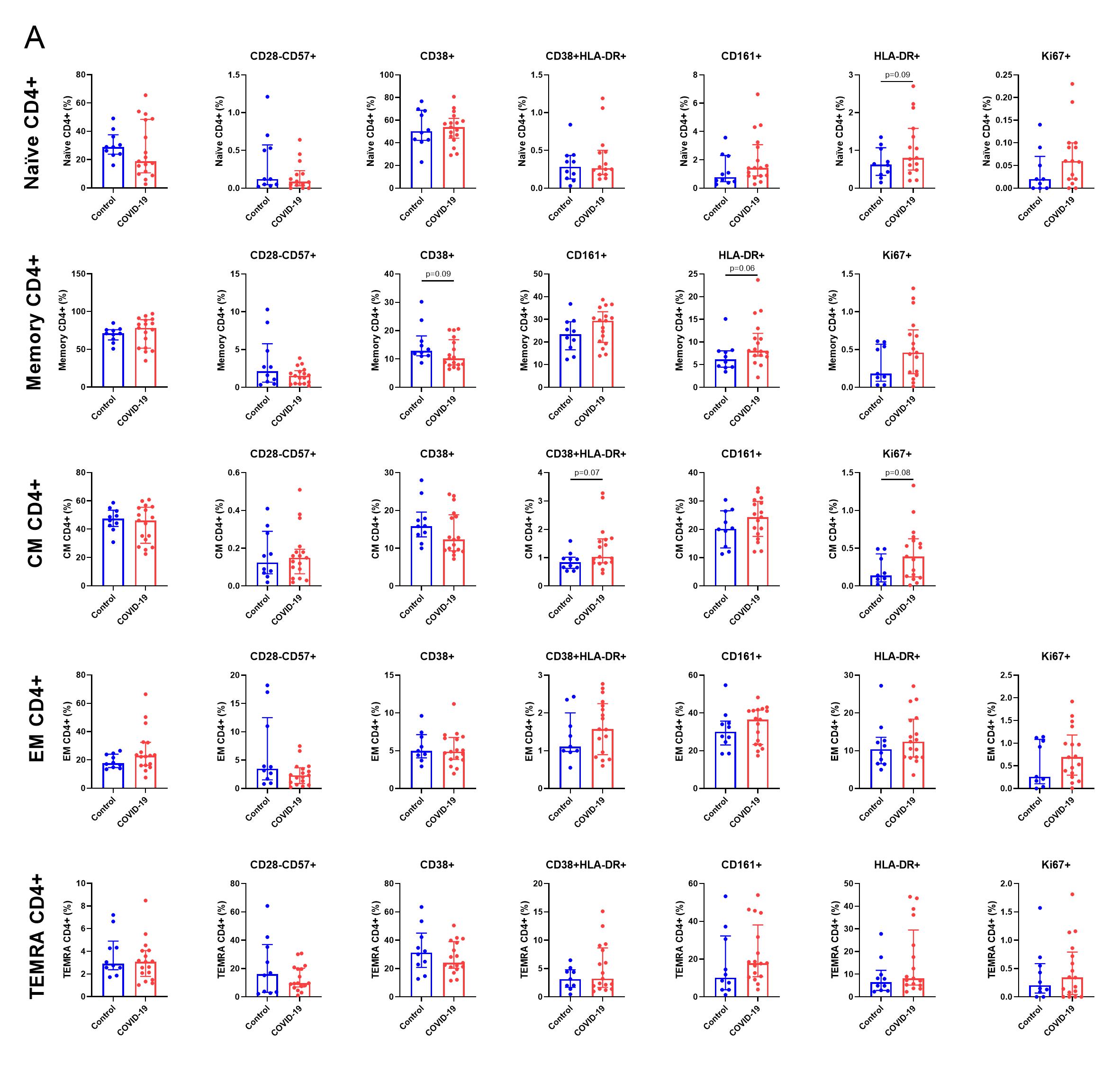

Supplement: Supplementary file 8 [file Image7.jpeg]

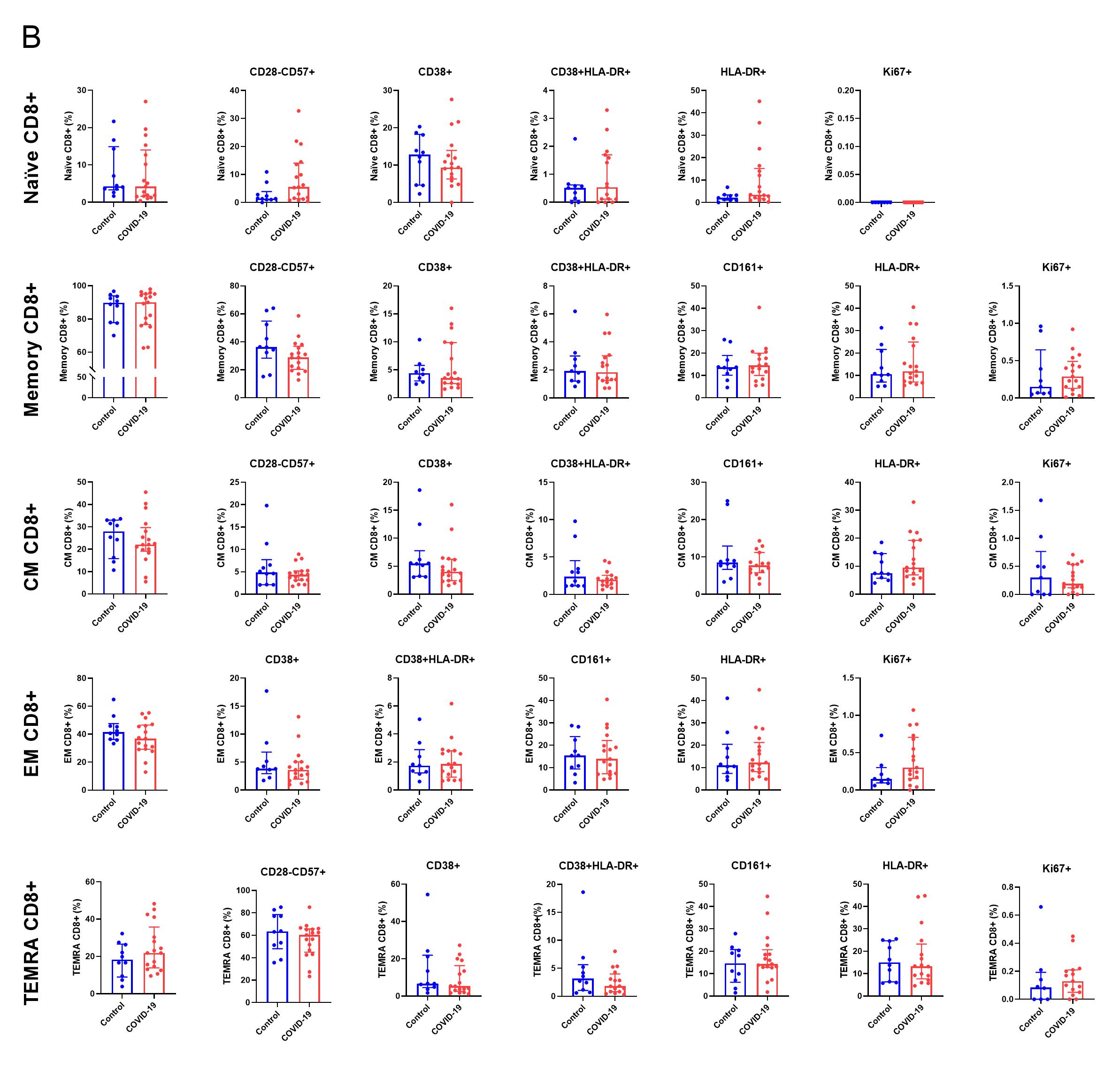

Supplement: Supplementary file 9 [file Image8.jpeg]

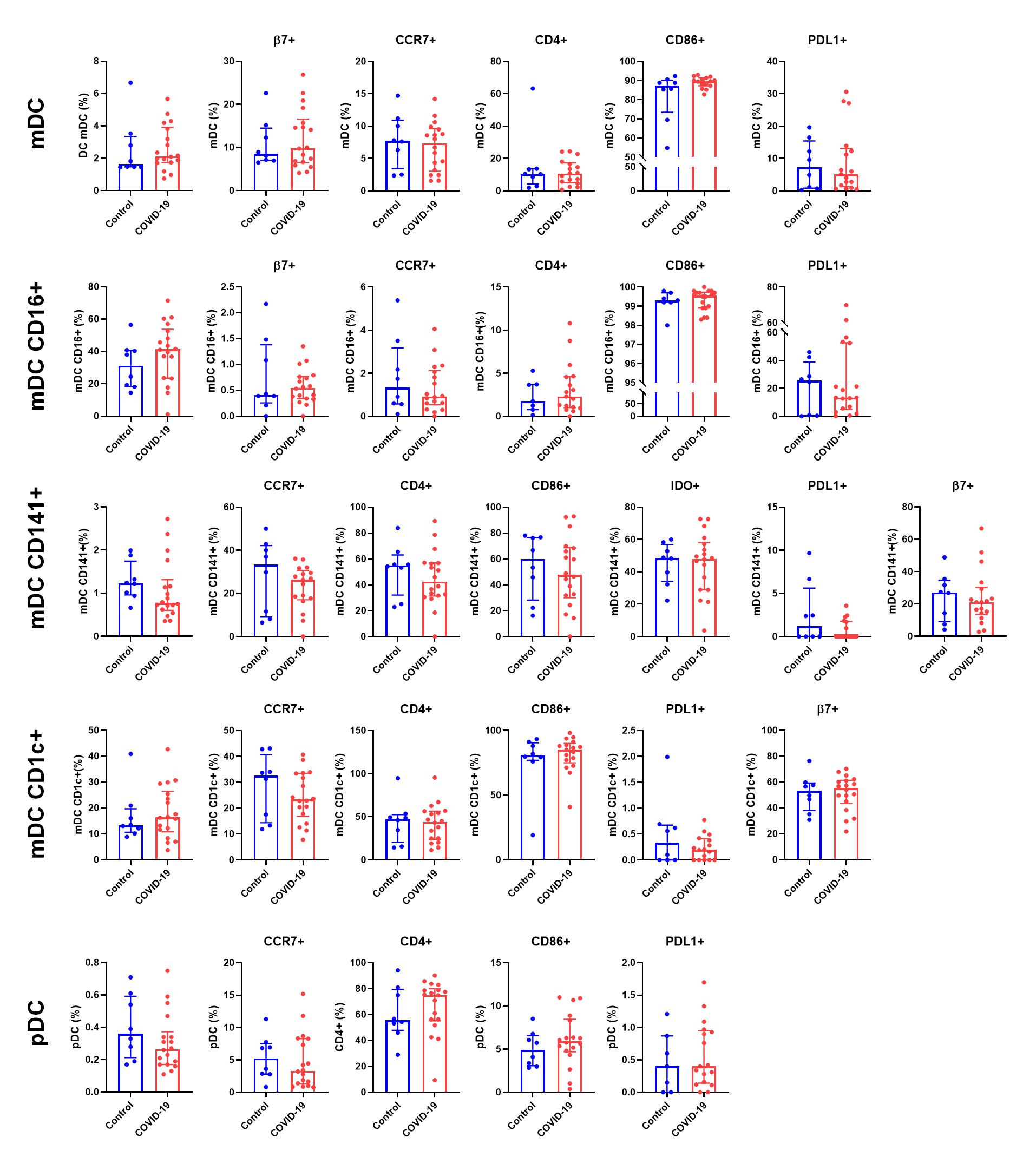

Supplement: Supplementary file 10 [file Image9.jpeg]
